# Tea Consumption and Liver Cancer: A Population-Based Case–Control Study in Eastern China

**DOI:** 10.3390/nu17162647

**Published:** 2025-08-15

**Authors:** Xing Liu, Aileen Baecker, Ming Wu, Jinyi Zhou, Ziyi Jin, Lina Mu, Na He, Jianyu Rao, Qing-Yi Lu, Liming Li, Jin-Kou Zhao, Zuo-Feng Zhang

**Affiliations:** 1 Department of Epidemiology, Fielding School of Public Health, University of California, Los Angeles, CA 90095 CA, USA; 2 Department of Epidemiology, School of Public Health, Fudan University, Shanghai 200032, China; 3 Department of Research & Evaluation, Kaiser Permanente Southern California, Pasadena, CA 91101, USA; 4 Jiangsu Provincial Center for Disease Control and Prevention, Nanjing 210009, China; 5 Department of Social and Preventive Medicine, School of Public Health and Health Professions, University at Buffalo, The State University of New York, Buffalo, NY 14214, USA; 6 Department of Pathology and Laboratory Medicine, UCLA David Geffen School of Medicine, Los Angeles, CA 90095, USA; 7 Department of Epidemiology, School of Public Health, Peking University, Beijing 100191, China; 8 Jonsson Comprehensive Cancer Center, University of California, Los Angeles, CA 90095, USA

**Keywords:** liver cancer, tea, interaction, case–control study, Chinese population

## Abstract

**Background/Objectives**: Identifying dietary factors influencing liver cancer is crucial for developing preventive measures. While tea polyphenols have demonstrated cancer-preventive activities in animal models, the evidence in humans is not definitive. This study aims to explore the association between tea consumption and liver cancer, as well as the interaction between tea drinking and other risk factors, in China, a country with a high incidence of liver cancer and substantial tea consumption. **Methods**: A population-based case–control study was conducted in Jiangsu Province from 2003 to 2010. Socio-demographic data, history of tea consumption, and serum markers of hepatitis B virus (HBV) and hepatitis C virus (HCV) infections were evaluated. Unconditional logistic regression was used to examine the associations between tea consumption and the odds of liver cancer. Potential interactions between tea consumption and other major liver cancer risk factors were assessed. **Results**: A total of 2011 incident liver cancer cases and 7933 controls were included in the analysis. Regular tea drinking showed an inverse association with the risk of liver cancer compared with those who never drank tea (OR: 0.79; 95% CI: 0.63–0.99). Current tea drinking showed an inverse association with liver cancer (OR: 0.51; 95% CI: 0.39–0.66), while former tea drinking showed a positive association (OR: 3.56; 95% CI: 2.42–5.23). Current tea consumption was inversely associated with liver cancer incidence among both hepatitis B surface antigen (HBsAg) positive (OR: 0.45; 95% CI: 0.28–0.73) and HBsAg negative participants (OR: 0.51, 95% CI: 0.36–0.73), among both never and ever tobacco smokers, ever alcohol drinkers (OR: 0.46; 95% CI: 0.33–0.63), and among those without family history of liver cancer. Multiplicative and additive interactions were observed between tea drinking and HBsAg, alcohol consumption, and history of raw water drinking. **Conclusions**: Tea consumption is inversely associated with the development of primary liver cancer, with potential interactions involving HBV infection, alcohol consumption, and raw (unsafe) water drinking. Increasing tea consumption—particularly among high-risk populations such as individuals who consume alcohol—may serve as an additional preventive measure for liver cancer. This should be considered alongside established strategies, including HBV vaccination, alcohol cessation, and avoidance of drinking raw water, to help reduce liver cancer risk.

## 1. Introduction

Liver cancer is the second leading cause of cancer-related death worldwide. Incidence is highest in East Asia and Africa, and China alone accounts for approximately half of all new cases and deaths every year [1]. The principal risk factors of liver cancer include chronic hepatitis B virus (HBV) infection, hepatitis C virus (HCV) infection, non-alcoholic fatty liver disease, excess alcohol consumption, cigarette smoking, and dietary exposure to aflatoxins. While these risk factors are well characterized, further research is needed to identify lifestyle and dietary strategies that can prevent the development of liver cancer.

Tea is the world’s most widely consumed beverage, traditionally prepared by infusing cured leaves of the Camellia sinensis shrub with hot water [2]. Although green tea was once confined largely to Asia, its popularity has surged globally in recent decades. The putative cancer-protective effects of green tea are attributed to its polyphenolic catechins-particularly epigallocatechin gallate (EGCG), epicatechin gallate (ECG), epigallocatechin (EGC), and epicatechin (EC) [3]. Because green tea undergoes minimal oxidation compared with black tea, its catechins retain stronger antioxidant activity [4]. Animal models and in vitro studies indicate that EGCG can suppress both the initiation and progression of tumors through its antioxidant and anti-inflammatory actions [5]. And epidemiological data links tea consumption to lower cancer risk via the same polyphenol-mediated mechanisms, including neutralizing reactive oxygen species, inducing apoptosis and suppressing receptor-dependent signaling pathways [6].

Evidence indicates a suggestive, though inconsistent, protective role of tea consumption against the risk of liver cancer [6]. A 2023 meta-analysis that included 14 cohort and 4 case–control studies found higher green tea consumption to be inversely associated with the risk of hepatocellular carcinoma (HCC) overall (RR, 0.80; 95% CI, 0.67–0.95). The association was significant in China (RR, 0.60; 95% CI, 0.39–0.92) but null in Japan (RR, 0.97; 95% CI, 0.81–1.16) [7]. Within that meta-analysis, the largest cohort—following 0.5 million Chinese adults and ascertaining 1874 incident liver cancer cases—reported a null association for green tea (RR, 0.98; 95% CI, 0.82, 1.18) [8]. Although a considerable amount of research has been conducted on this topic with no shortage of prospective cohort designs, the sample size of most studies is only in the hundreds, and adequate adjustment for important confounding factors such as HBV/HCV infection has only been carried out in some of them.

This study aims to address these inconsistencies by conducting a large-scale population-based case–control study in China, with comprehensive control for confounding factors. By focusing on a large sample size and detailed adjustment for major risk factors such as HBV/HCV infection, alcohol consumption, and tobacco smoking, this study seeks to provide more insights into the relationship between tea consumption and liver cancer risk. We hypothesize that tea drinking is inversely associated with the risk of primary liver cancer and may interact with major liver cancer risk factors.

## 2. Materials and Methods

### 2.1. Study Design

A population-based case–control study of risk factors for liver, esophageal, lung, and gastric cancers was conducted in four Jiangsu Province counties—Dafeng, Ganyu, Chuzhou, and Tongshan—between 2003 and 2010. The study design and methods have been described previously [9]. In brief, 2011 incident liver cancer cases and 7933 controls were identified from local cancer registries and county demographic records.

### 2.2. Participants

Liver cancer cases were identified from county-level cancer registries between 2003 and 2010. The inclusion criteria for the cases were the following: (1) age 18 years or above; (2) residence in the county for at least 5 years; and (3) diagnosis of primary liver cancer within the preceding 12 months. Population-based controls were randomly selected from demographic databases and matched to cases by age, sex, and county. Controls recruited for the four-cancer study were pooled to maximize statistical power [9]. Individuals previously diagnosed with any cancer were excluded. Participation rates were 37% among liver cancer cases and 87% among all controls.

### 2.3. Data Collection

The study was approved by the Institutional Review Board (IRB) of Jiangsu Provincial Health Department and the IRB of the University of California, Los Angeles (UCLA IRB-10-1501, renewed approval date: 21 July 2025). After obtaining informed consent, trained interviewers administered a standard questionnaire in a face-to-face interview to collect epidemiologic data on the following: (1) socio-demographic characteristics, (2) tea consumption, and (3) other liver cancer risk factors such as alcohol and tobacco use, possible exposure to mildew-contaminated food, and family history of liver cancer, etc.

### 2.4. Assessment of Tea Consumption

Participants were asked if they had regularly consumed tea, defined as tea drinking at least one cup per week for at least six months. Response options were the following: (1) “Yes, and still drinking”; (2) “Yes, but no longer drinking”; or (3) “No”. Those selecting option 1 were classified as current tea drinkers, option 2 as former tea drinkers, and option 3 as never tea drinkers. Current and former drinkers were classified as ever tea drinkers. In addition, we recorded age at first tea drinking, total years of consumption, tea type (green, black, flower, oolong, etc.), brew strength, daily frequency of adding fresh leaves or re-brewing, water temperature, water source (purified, tap, deep-well, shallow-well, rainwater, river, pond, or ditch), monthly tea-leaf consumption (g), and any change in tea drinking habits.

### 2.5. Assessment of Covariates

Socio-demographic characteristics including sex, age, county of residence, education, marital status, per-capita family income 10 years earlier, and BMI, etc., were collected via a questionnaire survey. “Ever-smoker” was defined as having smoked more than 100 cigarettes in a lifetime, and “ever-drinker” as having consumed alcohol occasionally or more frequently. Serum hepatitis B surface antigen (HBsAg) and HCV antibody were measured by an enzyme-linked immunosorbent assay (ELISA; Shanghai Kehua Diagnostic Medical Products Co., Ltd., Shanghai, China) at Jiangsu CDC according to the manufacturer’s protocol.

### 2.6. Statistical Analyses

Statistical analyses were performed using SAS 9.3 package (SAS Institute Inc., Cary, NC, USA). Categorical variables were compared with the chi-square test. Unconditional logistic regression was used to estimate crude odds ratios (cOR) and adjusted odd ratios (aOR) with 95% confidence intervals for liver cancer risk. The following potential confounding factors were adjusted in multiple logistic regression: age (continuous), sex (male = 0; female = 1), level of education (illiteracy = 0; primary = 1; middle school = 2; high school and college = 3), marital status (single, divorced, or widowed = 0; in marriage = 1), per-capita family income 10 years earlier (RMB Yuan/year, continuous), body mass index (BMI, continuous), family history of liver cancer (no = 0; yes = 1), pack-year of smoking (continuous), ethanol consumption (mL/week, continuous), HBsAg status (negative = 0; positive = 1), and country of residence (Dafeng = 1; Ganyu = 2; Chuzhou = 3; Tongshan = 4). A two-sided *p*-value less than 0.05 is considered as statistically significant. The Semi-Bayes (SB) method was employed to mitigate the risk of false positive findings by incorporating null priors (OR = 1.00; 95% prior limits: 0.25, 4), corresponding to the coefficients of mean zero and variance 0.5, thereby providing a more conservative and robust estimation of the associations [10].

Potential additive and multiplicative interactions were assessed using the relative excess risk due to interaction (RERI), the attributable proportion due to interaction (AP), the synergy index (S), and the ratio of the odds ratios (ROR) [11]. For these analyses, preventive factors were recoded, and the stratum with the lowest risk was set as the reference category to examine joint effects. Given the low prevalence of HCV infection in our study population, we did not adjust for HCV status in the multivariable regression models. Instead, we conducted a sensitivity analysis to further include HCV status.

## 3. Results

Table 1 shows the socio-demographic characteristics and tea consumption of the study population by cases and controls. Over 70% of participants were male, and the majority were over 60 years of age and married. HBsAg positivity was detected in 43.2% of the liver cancer cases and in 6.5% of the controls, while HCV antibody positivity was found in 0.9% of the cases and 0.8% of the controls. Among liver cancer cases, 80.6% reported never drinking tea, 9.2% reported being former tea drinkers, and 10.2% were current tea drinkers. Among the controls, 76.4% reported never drinking tea, 2.2% reported being former drinkers, and 21.5% were current tea drinkers. Green tea (76.6%) was the most consumed tea type in this population, followed by black tea (11.5%). Tea drinking habits varied by county of residence, sex, age, marriage status, and level of education. Additionally, tea drinkers were more likely to be alcohol drinkers and tobacco smokers.

Overall, ever drinking tea was inversely associated with liver cancer (cOR: 0.78; 95% CI: 0.69–0.88) (Table 2). This inverse association remained after adjusting for potential confounders (aOR: 0.79; 95% CI: 0.63–0.99) and after using semi-Bayes adjustment (SB-OR: 0.79; 95% PI: 0.64–0.99). Current tea drinking showed a stronger inverse association after adjustment for covariates (aOR: 0.51; 95% CI: 0.39–0.66), while former tea drinking was associated with an increased risk of liver cancer (aOR: 3.56; 95% CI: 2.42–5.23). Green tea (cOR: 0.78; 95% CI: 0.69–0.90) and black tea (cOR: 0.62; 95% CI: 0.44–0.88) showed inverse associations in the crude model, but these associations became marginal after a multivariable adjustment and semi-Bayes adjustment. No association was found between other kinds of tea drinking and liver cancer.

Those who reported monthly consumption of tea leaves less than 50 g showed an inverse association (aOR: 0.62; 95% CI: 0.40–0.96) compared with never drinkers, while no significant associations were found for those who consumed more (Table 2). Compared with never tea drinkers, those who reported drinking 1 cup of newly brewed tea leaves per day (aOR: 0.55; 95% CI: 0.38–0.78), those who re-brewed each cup of tea once or twice (aOR: 0.62; 95% CI: 0.41–0.94), and those who liked the light (aOR: 0.51; 95% CI: 0.31–0.83) or medium (aOR: 0.73; 95% CI: 0.55–0.98) concentration of tea showed significant inverse associations with liver cancer. There was no obvious association with further increased consumption of new tea leaves or increased times of brewing. Using spoiled water for tea brewing or boiling with tea leaves together (aOR: 0.78; 95% CI: 0.61–0.99) and drinking tea warm (aOR: 0.76; 95% CI: 0.59–0.98) appeared to show inverse associations, while brewing tea with warm water, drinking tea cold, hot, or very hot, did not.

**Figure 1:**
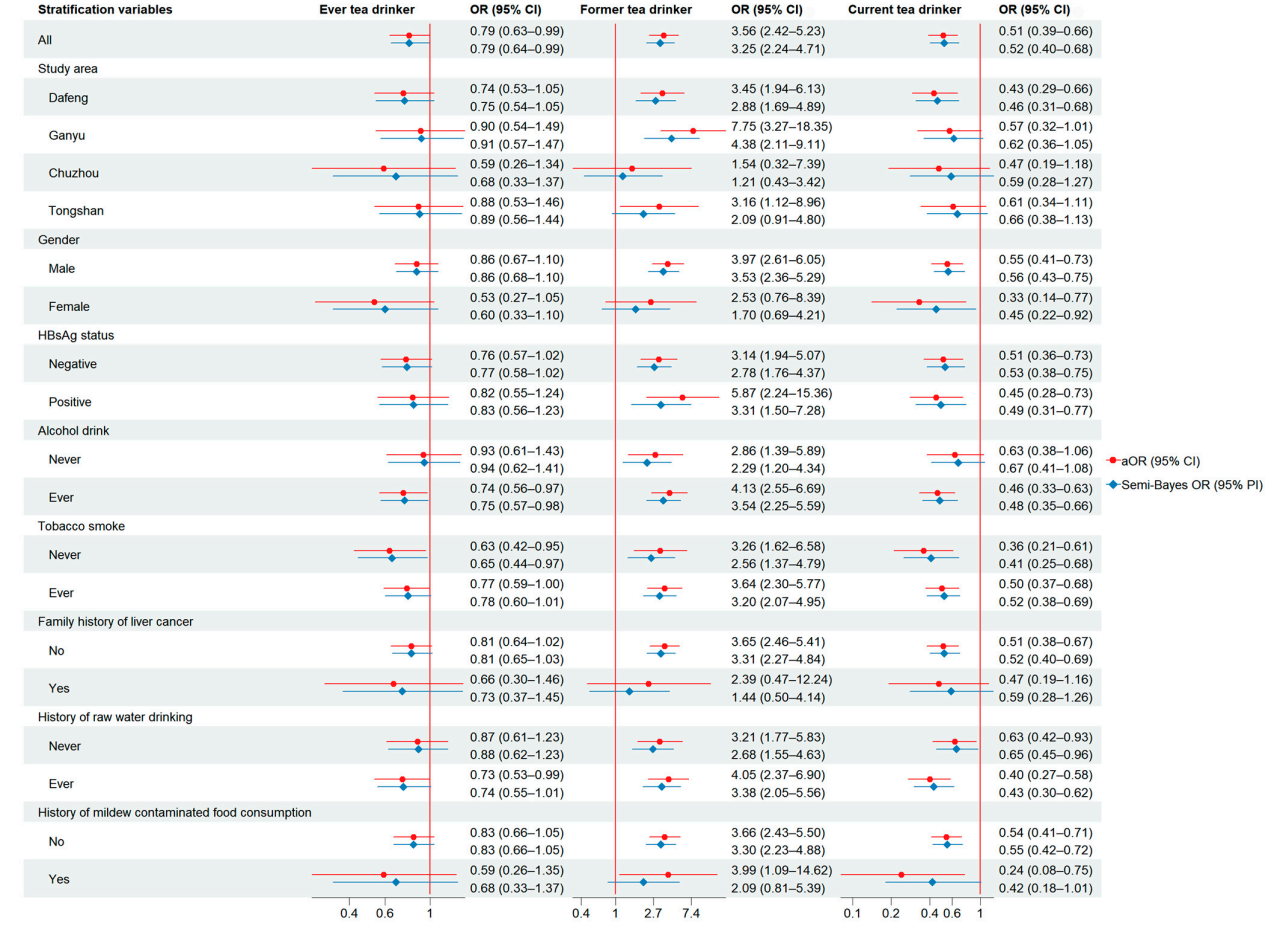
The association and semi-Bayes adjusted association between tea consumption and liver cancer stratified by major factors in Jiangsu Study 2003–2010. Adjusted for age (continuous), sex (male = 0; female = 1; except for stratified analyses by sex), education level (illiteracy = 0; primary = 1; middle school = 2; high school and college = 3), marital status (single, divorced, or widowed = 0; in marriage = 1), per-capita family income 10 years earlier (Yuan/year, continuous), BMI (continuous), family history of liver cancer (no = 0; yes = 1), weekly alcohol consumption in 1990s (continuous, mL/week), pack-year of smoking (continuous), HBsAg status (negative = 0; positive = 1), and county of residence (Dafeng = 1; Ganyu = 2; Chuzhou = 3; Tongshan = 4).

Figure 1 presents the associations between tea drinking and liver cancer stratified by sex and major risk factors, showing that the associations remained similar after stratifications. Current tea drinking appeared to have a stronger inverse association in women (aOR: 0.33; 95% CI: 0.14–0.77) than in men (aOR: 0.55; 95% CI: 0.41–0.73). Current tea drinking was inversely associated with liver cancer among both HBsAg positive (OR: 0.45; 95% CI: 0.28–0.73) and HBsAg negative participants (OR: 0.51; 95% CI: 0.36–0.73). A stronger inverse association was observed in those who reported ever drinking alcohol (aOR: 0.46; 95% CI: 0.33–0.63) compared with those who did not drink (aOR: 0.63; 95% CI: 0.38–1.06). Current tea drinking was protective among both never smokers (aOR: 0.36, 95% CI: 0.21–0.61) and ever smokers (aOR: 0.50, 95% CI: 0.37–0.68).

There was evidence of super-multiplicative (ROR: 1.78; 95% CI: 1.10–2.86) and super-additive (RERI: 0.45; 95% CI: 0.14–0.76) interaction between never tea drinking and alcohol drinking (Table 3) and of sub-multiplicative (ROR: 0.46; 95% CI: 0.29–0.72) interaction between never tea drinking and HBsAg positivity. Super-additive interaction was also observed between those who ever drank raw water and did not drink tea (RERI: 0.35; 95% CI: 0.10–0.61).

Since there were only 64 participants (11 of the liver cancer cases and 53 of the controls) who were positive for the HCV antibody, a sensitivity analysis was conducted to include HCV status in the multivariable regression model, and the results remained almost identical.

## 4. Discussion

In regions with a high incidence of liver cancer, such as China, identifying modifiable dietary factors is crucial for developing effective preventive strategies. This population-based case–control study aimed to investigate the association between tea consumption and liver cancer risk, as well as potential interactions with other major risk factors. The main findings revealed that regular tea consumption was inversely associated with the risk of liver cancer, with notable interactions involving hepatitis B virus (HBV) infection, alcohol consumption, and history of raw water drinking.

The current study identified a significant inverse association, between regular tea consumption and liver cancer (aOR: 0.79, 95% CI: 0.63, 0.99), which was largely driven by the consumption of green tea. Inverse associations were observed among individuals who consumed 1 cup of fresh tea daily, those who consumed 1–50 g of tea leaves monthly, and those who re-brewed tea (refilled with water) 1–2 times per day. However, drinking more than one cup of tea per day, consuming more tea leaves, or re-brewing tea from the same teapot more times did not confer additional benefits. Additionally, individuals who drank more concentrated tea did not exhibit a stronger inverse association compared to those who consumed tea of lighter or medium concentration. In sum, we found that ever tea consumption was associated with an approximately 20% decreased risk of liver cancer, but no significant dose–response relationship was observed. An earlier meta-analysis of prospective cohort studies in Asian populations reported a significant association between green tea consumption and reduced risk of liver cancer (RR: 0.88; 95% CI: 0.81; 0.97), but the association was only marginally significant for one additional daily cup of tea (RR: 0.97; 95% CI: 0.95, 1.00) [12]. The China Kadoorie Biobank (CKB) study followed-up on 455,981 participants for 10 years and reported a null association between tea drinking and liver cancer risk when compared to daily consumers who added tea leaves > 4.0 g/day vs. less-than-weekly consumers (HR: 1.08; 95% CI: 0.75–1.55) [8]. One possible explanation for the difference may be that the comparisons in the current study were all made relative to never tea drinkers, whereas the CKB study used less-than-weekly consumers as the reference group. Given the inverse association observed at moderate consumption levels in the current study, the association may be attenuated when the reference group included occasional tea drinkers in CKB.

In this study, individuals who reported themselves as former tea drinkers (3% of cases and 1.6% of controls) exhibited higher odds of liver cancer. Moreover, a decrease in tea consumption over time was also associated with increased odds of liver cancer. These positive associations may be influenced by reverse causality, as individuals with liver cancer or its precursor diseases often experience symptoms such as lack of appetite and discomfort in the stomach, which could lead them to reduce their tea consumption over time. Future research should consider more detailed dietary assessments and longitudinal data to better account for changes in tea consumption patterns over time.

For a long time, green tea has attracted extensive research attention for its potential anti-cancer effects, including against liver cancer, due to its rich antioxidant components such as polyphenols and epigallocatechin gallate (EGCG). Studies suggest that active compounds in green tea, such as EGCG, may inhibit the initiation and development of cancer through mechanisms like regulating proliferation and apoptosis, inhibiting tumor metastasis and angiogenesis [13,14]. Extensive epidemiological studies support the anti-cancer effects of green tea, but research linking specific anti-cancer substances within it and corresponding brewing methods to tumors in population studies is still very limited [15,16]. We were also interested in examining whether the temperature of the water used to brew tea and the temperature at which tea is consumed are associated with liver cancer risk. We found that using boiling water to brew tea was inversely associated with liver cancer risk, while using warm water showed no association. The temperature of water used for brewing tea significantly impacts the release and degradation of bioactive compounds [17]. Boiling water maximizes the extraction of caffeine, amino acids, and catechins in green tea, while fully releasing polyphenols and aroma compounds in black, oolong, and pu-erh teas. However, high temperatures accelerate the degradation of heat-sensitive nutrients, such as vitamin C and EGCG, which isomerizes into less active GCG at ≥95 °C [18,19]. Regarding the temperature at which tea is consumed, we found that drinking warm tea was inversely associated with liver cancer risk, whereas drinking cold or hot/very hot tea showed no association. The mechanisms linking tea temperature to liver cancer remain unclear and warrant further investigation.

The large sample size, comprehensive questionnaire survey, and laboratory tests enabled a confounding control, stratified analysis, and test for potential interactions in this study. The inverse associations remained stable in stratified analyses by major risk factors, including HBV infection, tobacco smoking, and history of raw water or mildew contaminated food intake, and was significant among alcohol drinkers. The observed interactions between never tea drinking and alcohol drinking (super-additive and super-multiplicative), never tea drinking and HBsAg positivity (sub-multiplicative), as well as ever drinking raw water and not drinking tea (super-additive), highlight the complex interplay of lifestyle factors and health outcomes. The super-additive interactions may be biologically plausible given tea’s potential protective effects, such as its antioxidant and anti-inflammatory properties, which can mitigate the adverse effects of alcohol and contaminated water. Green tea may protect against liver cancer in alcohol drinkers by reducing oxidative stress and inflammation, enhancing detoxification, inhibiting cancer cell growth, and modulating inflammatory pathways, primarily through its polyphenols such as catechin, which counteract alcohol-induced liver damage [20,21]. Conversely, the sub-multiplicative interaction between never tea drinking and HBsAg positivity indicates that the combined effect of these two factors is less than expected based on their individual contributions, suggesting that never tea drinking might not further increase the risk associated with HBsAg positivity. One Chinese case–control study found an increased risk among non-green tea drinkers with HBV/HCV-infected individuals [22]. In contrast, a Japanese cohort study found no association for green tea overall or in those HBV/HCV-infected participants [23]. The limited sample size, variations in tea brewing methods, and different predominant hepatitis virus infections (HCV in Japan and HBV in China) may account for the observed differences in both the overall and stratified analyses [7,24,25]. These findings underscore the importance of considering multiple factors and their interplay in understanding and preventing disease. Future research should further explore the mechanisms underlying these interactions to better understand their health implications.

Limitations of this study include a relatively low responding rate among the cases. Liver cancer is highly fatal, and most of the non-responders were in poor health or even deceased when we identified them from the cancer registry and reached out to them for an interview and blood draw. The second limitation was the case–control design, where disease status was determined before measurement of risk factors. This may have affected the associations for a small percentage of cases who self-reported being former tea drinkers or who reported drinking less tea in recent years. Because tea drinking is not a known risk factor or protective factor for liver cancer, recall bias should not be a major threat to the validity. Ever tea drinking was still found to be protective after combining former tea drinkers with current tea drinkers. Third, the measurement of tea drinking was based on self-report using a questionnaire in a retrospective manner, which could be compromise precision. However, since tea drinking is usually a long-term and stable habit, the self-reported amount, concentration, and duration of tea consumption should be reliable.

## 5. Conclusions

Given the high incidence and mortality of liver cancer in China, the development of effective prevention strategies is of utmost importance. While controlling HBV and HCV infections, reducing alcohol consumption, and decreasing cigarette smoking are established and effective preventive measures, complementary dietary and nutritional strategies hold significant promise. In this study, we found that regular and moderate tea intake showed an inverse association with the risk of liver cancer. Moreover, tea consumption demonstrated a preventive association with liver cancer that could be integrated into comprehensive prevention programs targeting major liver cancer risk factors, including HBV infection and alcohol drinking. Future research should further explore the underlying mechanisms and optimize the application of tea consumption in liver cancer prevention strategies.

## Figures and Tables

**Table 1:** Socio-demographic characteristics of subjects in Jiangsu liver cancer study 2003–2010 (N = 9944).

Characteristics	Case (*n*, %)	Tea Consumption (%)			Control (*n*, %)	Tea Consumption (%)			χ^2^	p *
		Never	Former	Current		Never	Former	Current		
Total	2011	80.6	9.2	10.2	7933	76.4	2.2	21.5		
County of residence									42.271	<0.001
Dafeng	632 (31.4)	80.5	8.7	10.8	2534 (31.9)	73.1	3.1	23.8		
Ganyu	390 (19.4)	55.6	21.5	22.8	2010 (25.3)	58.6	2.4	39.0		
Chuzhou	301 (15.0)	81.0	10.0	9.0	1134 (14.3)	77.7	2.8	19.5		
Tongshan	688 (34.2)	94.6	2.2	3.2	2255 (28.5)	95.3	0.6	4.2		
Sex									15.441	<0.001
Male	1534 (76.3)	75.7	11.6	12.7	5705 (71.9)	70.3	2.7	26.9		
Female	477 (23.7)	96.2	1.3	2.5	2228 (28.1)	91.7	0.8	7.5		
Age group (years)									338.90	<0.001
<50	471 (23.4)	84.1	8.3	7.6	872 (11.0)	78.0	0.7	21.3		
50–59	603 (30.0)	79.9	8.8	11.3	1773 (22.4)	74.9	2.4	22.7		
60–69	515 (25.6)	78.6	10.9	10.5	2542 (32.0)	76.2	1.9	22.0		
70–79	322 (16.0)	77.6	10.3	12.1	2168 (27.3)	75.5	3.0	21.5		
80+	100 (5.0)	88.0	3.0	9.0	578 (7.3)	82.4	2.3	15.4		
In marriage									22.886	<0.001
Yes	1717 (86.0)	80.6	9.3	10.1	6426 (81.5)	75.4	2.2	22.4		
No	279 (14.0)	81.0	7.9	11.1	1463 (18.5)	80.4	2.0	17.6		
Missing	15				44					
Education level									81.962	<0.001
Illiteracy	764 (38.2)	83.3	8.5	8.3	3796 (47.9)	80.1	1.8	18.2		
Primary	662 (33.1)	80.4	9.5	10.1	2490 (31.5)	74.1	2.7	23.3		
Middle	461 (23.1)	79.6	10.0	10.4	1298 (16.4)	72.5	2.5	25.0		
High	101 (5.0)	70.3	6.9	22.8	292 (3.7)	67.1	2.4	30.5		
College	12 (0.6)	58.3	16.7	25.0	40 (0.5)	52.5	2.5	45.0		
missing	11				17					
Per-capita family income 10 years earlier (RMB yuan/year)									5.849	0.119
<1000	392 (20.0)	80.6	10.2	9.2	1656 (21.3)	76.0	2.2	21.7		
1000–1499	405 (20.6)	79.5	8.4	12.1	1512 (19.5)	78.4	1.7	20.0		
1500–2499	487 (24.8)	82.3	10.3	7.4	2057 (26.5)	77.8	2.2	20.0		
2500+	680 (34.6)	79.9	7.7	12.5	2542 (32.7)	74.6	2.4	23.0		
missing	47				166					
BMI									122.36	<0.001
<18.5	211 (10.7)	81.0	7.1	11.9	452 (5.7)	79.9	1.3	18.8		
18.5–23.9	1315 (66.4)	80.3	10.2	9.5	4791 (60.7)	76.2	2.1	21.7		
24.0–27.9	379 (19.1)	83.1	7.4	9.5	2198 (27.9)	77.1	2.3	20.6		
28.0+	75 (3.8)	65.3	8.0	26.7	449 (5.7)	71.5	3.1	25.4		
missing	31				43					
HBsAg									1273.0	<0.001
Negative	689 (56.8)	89.3	4.5	6.2	6074 (93.5)	77.2	2.0	20.8		
Positive	524 (43.2)	79.4	9.4	11.3	425 (6.5)	78.6	1.7	19.8		
missing	798				1434					
Ever alcohol drinker									59.407	<0.001
No	885 (44.0)	91.8	3.2	5.1	4254 (53.6)	88.9	1.4	9.7		
Yes	1126 (56.0)	71.9	13.9	14.3	3679 (46.4)	61.8	3.1	35.0		
Ever tobacco smoker									17.231	<0.001
No	971 (48.3)	91.7	4.3	4.0	4241 (53.5)	88.1	1.2	10.7		
Yes	1040 (51.7)	70.3	13.7	16.1	3692 (46.5)	62.8	3.3	33.9		
Family history of liver cancer									359.49	<0.001
No	1735 (86.3)	80.4	9.3	10.3	7684 (96.9)	76.5	2.2	21.3		
Yes	276 (13.7)	81.9	8.0	10.1	249 (3.1)	73.1	1.6	25.3		

* Statistical tests compared the differences in the distribution of characteristics between cases and controls.

**Table 2:** Crude, adjusted, and semi-Bayes associations between tea drinking and liver cancer in Jiangsu Study 2003–2010.

Tea Drinking Patterns	Cases	Controls	cOR (95% CI)	aOR (95% CI) *	SB-aOR (95% CI)
Ever tea drinker					
No	1621	6057	1.00	1.00	1.00
Yes	390	1876	0.78 (0.69–0.88)	0.79 (0.63–0.99)	0.79 (0.64–0.99)
Regular tea drinker					
No	1621	6057	1.00	1.00	1.00
Former	184	173	3.97 (3.21–4.93)	3.56 (2.42–5.23)	3.25 (2.24–4.71)
Current	206	1703	0.45 (0.39–0.53)	0.51 (0.39–0.66)	0.52 (0.40–0.68)
Tea type					
Never drink	1621	6057	1.00	1.00	1.00
Green	301	1434	0.78 (0.69–0.90)	0.80 (0.62–1.04)	0.81 (0.63–1.04)
Black	37	223	0.62 (0.44–0.88)	0.65 (0.39–1.10)	0.69 (0.42–1.11)
Flower	27	81	1.25 (0.80–1.93)	0.96 (0.47–1.96)	0.97 (0.51–1.83)
Oolong	2	22	0.34 (0.08–1.45)	1.34 (0.25–7.11)	1.13 (0.39–3.28)
Other	19	97	0.73 (0.45–1.20)	0.85 (0.39–1.85)	0.88 (0.45–1.74)
Monthly tea-leaf consumption (g)					
Never drink	1621	6057	1.00	1.00	1.00
1–50	51	406	0.47 (0.35–0.63)	0.62 (0.40–0.96)	0.65 (0.43–0.98)
51–100	103	481	0.80 (0.64–1.00)	0.81 (0.56–1.17)	0.82 (0.58–1.17)
101–200	88	374	0.88 (0.69–1.12)	0.89 (0.58–1.36)	0.90 (0.60–1.35)
200+	135	550	0.92 (0.75–1.12)	0.90 (0.61–1.33)	0.91 (0.62–1.32)
Daily frequency of adding fresh leaves					
Never drink	1621	6057	1.00	1.00	1.00
1	106	643	0.62 (0.50–0.76)	0.55 (0.38–0.78)	0.57 (0.40–0.81)
2	172	766	0.84 (0.71–1.00)	0.98 (0.72–1.32)	0.98 (0.73–1.32)
3	66	306	0.81 (0.61–1.06)	0.88 (0.53–1.46)	0.89 (0.56–1.44)
4+	40	131	1.14 (0.80–1.63)	1.25 (0.63–2.46)	1.20 (0.65–2.21)
Daily frequency of re-brewing					
Never drink	1621	6057	1.00	1.00	1.00
1~2	87	404	0.81 (0.63–1.02)	0.62 (0.41–0.94)	0.64 (0.43–0.96)
3+	294	1438	0.76 (0.67–0.88)	0.86 (0.67–1.10)	0.86 (0.68–1.10)
Brew strength					
Never drink	1621	6057	1.00	1.00	1.00
Light	61	378	0.60 (0.46–0.80)	0.51 (0.31–0.83)	0.55 (0.35–0.87)
Medium	202	974	0.78 (0.66–0.91)	0.73 (0.55–0.98)	0.74 (0.56–0.98)
Strong	126	506	0.93 (0.76–1.14)	1.18 (0.84–1.67)	1.17 (0.84–1.63)
Water temperature					
Never drink	1621	6057	1.00	1.00	1.00
Warm	66	353	0.70 (0.53–0.91)	0.88 (0.57–1.37)	0.89 (0.59–1.35)
Spoiled or boil together	317	1499	0.79 (0.69–0.90)	0.78 (0.61–0.99)	0.79 (0.62–1.00)
Temperature of tea when drink					
Never drink	1621	6057	1.00	1.00	1.00
Cold	8	61	0.49 (0.23–1.03)	0.27 (0.07–1.02)	0.51 (0.19–1.33)
Warm	251	1308	0.72 (0.62–0.83)	0.76 (0.59–0.98)	0.77 (0.60–0.98)
Hot/very hot	127	476	1.00 (0.81–1.22)	1.03 (0.70–1.50)	1.03 (0.71–1.48)
Water source					
Never drink	1621	6057	1.00	1.00	1.00
Tap or purified	171	792	0.81 (0.68–0.96)	0.82 (0.60–1.13)	0.83 (0.61–1.13)
Deep-well	75	504	0.56 (0.43–0.71)	0.47 (0.31–0.70)	0.50 (0.34–0.74)
Shallow-well	130	475	1.02 (0.84–1.25)	1.59 (1.08–2.33)	1.54 (1.06–2.23)
Pool/ditch/river/rain water	12	79	0.57 (0.31–1.04)	0.33 (0.11–1.02)	0.51 (0.21–1.21)
Total years of tea consumption					
Never drink	1621	6057	1.00	1.00	1.00
<30	214	968	0.83 (0.71–0.97)	0.69 (0.53–0.91)	0.70 (0.54–0.91)
30+	171	875	0.73 (0.61–0.87)	1.02 (0.74–1.41)	1.02 (0.74–1.39)

* Adjusted for age (continuous), sex (male = 0; female = 1), education level (illiteracy = 0; primary = 1; middle school = 2; high school and college = 3), marital status (single, divorced, or widowed = 0; in marriage = 1), per-capita family income 10 years earlier (Yuan/year, continuous), BMI (continuous), family history of liver cancer (no = 0; yes = 1), weekly alcohol consumption in 1990s (continuous, mL/week), pack-year of smoking (continuous), HBsAg status (negative = 0; positive = 1), and county of residence (Dafeng = 1; Ganyu = 2; Chuzhou = 3; Tongshan = 4).

**Table 3:** Interaction between ever tea drinking regularly and other risk factors on liver cancer in Jiangsu Study 2003–2010 (N = 9944).

Variables	Ever Drink Tea	Case/Control	Crude OR (95% CI)	Adjusted OR (95% CI) *	SB-Adjusted OR (95% PI)	Interaction	
HBsAg positive							
No	Yes	74/1387	1.00	1.00	1.00	ROR	0.46 (0.29–0.72)
No	No	615/4687	2.46 (1.92–3.15)	1.66 (1.25–2.22)	1.63 (1.23–2.15)	RERI	−5.09 (−11.70–1.53)
Yes	Yes	108/91	22.25 (15.46–32.01)	18.65 (12.42–28.00)	14.79 (10.02–21.85)	AP	−0.36 (−0.84–0.12)
Yes	No	416/334	23.35 (17.74–30.72)	14.22 (10.41–19.44)	12.51 (9.22–16.96)	S	0.72 (0.50–1.05)
Ever drink alcohol							
No	Yes	73/472	1.00	1.00	1.00	ROR	1.78 (1.10–2.86)
No	No	812/3782	1.39 (1.07–1.80)	0.86 (0.57–1.31)	0.87 (0.58–1.30)	RERI	0.45 (0.14–0.76)
Yes	Yes	317/1404	1.46 (1.11–1.92)	1.20 (0.77–1.87)	1.18 (0.77–1.80)	AP	0.20 (0.04–0.35)
Yes	No	809/2275	2.30 (1.77–2.98)	1.83 (1.21–2.77)	1.74 (1.17–2.59)	S	1.53 (0.95–2.48)
Ever smoke							
No	Yes	81/503	1.00	1.00	1.00	ROR	1.11 (0.70–1.75)
No	No	890/3738	1.48 (1.16–1.89)	1.28 (0.86–1.92)	1.26 (0.85–1.85)	RERI	0.30 (−0.18–0.78)
Yes	Yes	309/1373	1.40 (1.07–1.82)	1.40 (0.91–2.15)	1.36 (0.90–2.05)	AP	0.15 (−0.12–0.42)
Yes	No	731/2319	1.96 (1.53–2.51)	1.98 (1.33–2.95)	1.88 (1.28–2.76)	S	1.44 (0.59–3.53)
Family history of liver cancer							
No	Yes	340/1809	1.00	1.00	1.00	ROR	1.21 (0.62–2.37)
No	No	1395/5875	1.26 (1.11–1.44)	1.24 (0.98–1.56)	1.23 (0.98–1.55)	RERI	1.55 (−1.00–4.10)
Yes	Yes	50/67	3.97 (2.70–5.83)	3.57 (1.96–6.48)	2.92 (1.69–5.06)	AP	0.29 (−0.14–0.72)
Yes	No	226/182	6.61 (5.27–8.29)	5.36 (3.74–7.67)	4.82 (3.41–6.83)	S	1.55 (0.69–3.48)
Possibility of mildew contamination on food							
No	Yes	344/1672	1.00	1.00	1.00	ROR	1.41 (0.66–3.05)
No	No	1414/5534	1.24 (1.09–1.41)	1.21 (0.96–1.53)	1.20 (0.96–1.51)	RERI	0.41 (−0.11–0.92)
Yes	Yes	44/181	1.18 (0.83–1.68)	0.92 (0.45–1.87)	0.94 (0.50–1.76)	AP	0.22 (−0.04–0.49)
Yes	No	175/465	1.83 (1.49–2.26)	1.57 (1.10–2.23)	1.53 (1.08–2.15)	S	1.96 (0.66–5.83)
Ever drink raw water							
No	Yes	177/933	1.00	1.00	1.00	ROR	1.41 (0.93–2.14)
No	No	535/2595	1.09 (0.90–1.31)	1.01 (0.73–1.39)	1.01 (0.74–1.38)	RERI	0.35 (0.10–0.61)
Yes	Yes	204/896	1.20 (0.96–1.50)	0.98 (0.67–1.43)	0.98 (0.68–1.41)	AP	0.22 (0.05–0.39)
Yes	No	1034/3320	1.64 (1.38–1.96)	1.40 (1.04–1.90)	1.38 (1.03–1.85)	S	2.24 (0.71–7.07)

* Adjusted for age (continuous), sex (male = 0; female = 1; except for stratified analyses by sex), education level (illiteracy = 0; primary = 1; middle school = 2; high school and college = 3), marital status (single, divorced, or widowed = 0; in marriage = 1), per-capita family income 10 years earlier (Yuan/year, continuous), BMI (continuous), family history of liver cancer (no = 0; yes = 1), weekly alcohol consumption in 1990s (continuous, mL/week), pack-year of smoking (continuous), HBsAg status (negative = 0; positive = 1) and county of residence (Dafeng = 1; Ganyu = 2; Chuzhou = 3; Tongshan =4). For alcohol drinking, mildew contamination, and raw-water-drinking history, their interactions on an additive scale were examined using the crude ORs, since the adjusted ones are not at the same direction. The sample size in analyses for interaction may vary due to different numbers of missing for each variable.

## Data Availability

The original contributions presented in the study are included in the article, further inquiries can be directed to the corresponding author.

## Abbreviations

The following abbreviations are used in this manuscript:

HBV	Hepatitis B virus
HCV	Hepatitis C virus
HBsAg	Hepatitis B surface antigen
EGCG	Epigallocatechin gallate
ECG	Epicatechin gallate
EGC	Epigallocatechin
EC	Epicatechin
HCC	Hepatocellular carcinoma
ELISA	Enzyme-linked immunosorbent assay
BMI	Body mass index
SB	Semi-Bayes
RERI	Relative excess risk due to interaction
AP	Attributable proportion due to interaction
S	Synergy index
ROR	Ratio of the odds ratios
CKB	China Kadoorie Biobank
GCG	Gallocatechin gallate
